# Critical Updates on Chronic Hepatitis B Virus Infection in 2021

**DOI:** 10.7759/cureus.19152

**Published:** 2021-10-30

**Authors:** Cyriac A Philips, Rizwan Ahamed, Jinsha K Abduljaleel, Sasidharan Rajesh, Philip Augustine

**Affiliations:** 1 Clinical and Translational Hepatology, The Liver Institute, Rajagiri Hospital, Aluva, IND; 2 Gastroenterology and Advanced Gastrointestinal Endoscopy, Center of Excellence in Gastrointestinal Sciences, Rajagiri Hospital, Aluva, IND; 3 Diagnostic and Interventional Radiology, Center of Excellence in Gastrointestinal Sciences, Rajagiri Hospital, Aluva, IND

**Keywords:** acute hepatitis, liver failure, hepatocellular carcinoma, chronic hepatitis, aclf, acute liver failure, antiviral, portal hypertension, cirrhosis, hbv

## Abstract

Chronic hepatitis B virus (HBV) infection is a global healthcare burden in the form of chronic liver disease, cirrhosis, liver failure and liver cancer. There is no definite cure for the virus and even though extensive vaccination programs have reduced the burden of liver disease in the future population, treatment options to eradicate the virus from the host are still lacking. In this review, we discuss in detail current updates on the structure and applied biology of the virus in the host, examine updates to current treatment and explore novel and state-of-the-art therapeutics in the pipeline for management of chronic HBV. Furthermore, we also specifically review clinical updates on HBV-related acute on chronic liver failure (ACLF). Current treatments for chronic HBV infection have seen important updates in the form of considerations for treating patients in the immune tolerant phase and some clarity on end points for treatment and decisions on finite therapy with nucleos(t)ide inhibitors. Ongoing cutting-edge research on HBV biology has helped us identify novel target areas in the life cycle of the virus for application of new therapeutics. Due to improvements in the area of genomics, the hope for therapeutic vaccines, vector-based treatments and focused management aimed at targeting host integration of the virus and thereby a total cure could become a reality in the near future. Newer clinical prognostic tools have improved our understanding of timing of specific treatment options for the catastrophic syndrome of ACLF secondary to reactivation of HBV. In this review, we discuss in detail pertinent updates regarding virus biology and novel therapeutic targets with special focus on the appraisal of prognostic scores and treatment options in HBV-related ACLF.

## Introduction and background

The prevalence of hepatitis B virus (HBV) surface antigen positivity among the general population differs according to geographical region, which also dictates possible routes of transmission. In low prevalence (<2%) regions such as North America and Western Europe, age at infection in early adulthood and route of transmission is mostly sexual and percutaneous. In regions of moderate prevalence (2-8%), age at infection in childhood and perinatal transmission is the most common mode of spread. Previously, age-dependent phases of HBV were described as immunotolerant phase (high replication, low-inflammation), immunoactive phase, inactive carrier state (low replication levels with normal/nearly normal serum aminotransferase levels) and reactivation. Nonetheless, these have been renamed recently as HBV envelope antigen (HBeAg)-positive infection, HBeAg-positive hepatitis and HBeAg-negative infection and HBeAg-negative hepatitis. Progression to cirrhosis in HBeAg-positive patients occurs at a rate of 2 to 5.5% per year increasing from 8 to 20% in five years. Inactive carriers who have normal aminotransferase levels and HBV DNA levels <2,000 IU/ml experience disease regression at the rate of 0.5 to 2% per year. HBeAg-negative hepatitis, or the reactivation phase, represents a progressive stage of chronic HBV. Anti-HBe (antibody to e-antigen)-positive patients experience rapid progression to cirrhosis at an annual rate of 8 to 20%. Patients with cirrhosis progress to advanced liver disease and hepatic failure at a rate of 16% over five years [1-4]. Chronic HBV infection remains a huge burden on the patients, their family and the healthcare system the world over, mostly in the Asia-Pacific region. There have been rapid developments toward a functional cure of HBV infection, with novel compounds currently in various study phases. Our current understanding of pathogenesis, immunology and clinical outcomes of HBV infection has seen vast updates over the last decade. In this narrative review, we provide in-depth discussions on the current understanding of biology and immuno-pathogenesis; variants and genotypes of HBV infection and extrapolate the same toward discussing novel therapies. We also explore current treatment options and discuss with clarity the guideline recommendations on HBV treatments, specifically updates on the special clinical condition of acute on chronic liver failure (ACLF) related to HBV infection.

## Review

Updates on HBV-related applied biology

The HBV Structure

HBV infection is a dynamic disease that encompasses biochemical, histological and clinical changes that occur over time, depending on the mode of acquisition, host and environmental factors. Within the host, HBV can exist in three forms, the infectious virion (Dane particle) and non-infectious particles that include enveloped nucleocapsids containing immature DNA/RNA, subviral particles (spheres, filaments lacking nucleocapsid proteins) and naked nucleocapsids [5,6]. According to the Baltimore Classification, a system utilized to group viruses taking into consideration both transcription and replication, on the basis of manner of messenger RNA (mRNA) synthesis, HBV belongs to Group VII which includes double-stranded DNA viruses with an RNA intermediate. HBV is a partially double-stranded hepadnavirus with a size of 42 nm containing a relaxed-circular DNA (rcDNA) genome with complete minus and incomplete plus strands. It has a host-derived outer surface lipid coat containing surface antigen which consists of large (L-), middle (M-) and small (S-HBsAg) and an inner core protein called the hepatitis B core antigen (HBcAg). The pre-S1 domain of the L-HBs plays a key role in viral envelopment and drives infectivity [7].

The viral genome encodes four overlapping open reading frames (ORFs): C (pre-core and core regions), P, S (pre-S1, pre-S2, S regions), and X (from which functional viral proteins are produced). The core antigen (nucleocapsid) protein, HBcAg; and the ‘e’ antigen (HBeAg) and 22-kDa pre-core protein (p22cr) are produced from ORF-C core and pre-core regions respectively. The polymerase protein (Pol) encoded from ORF-P is made of terminal domain with functions of encapsidation and initiation of minus strand synthesis; the reverse transcriptase domian (RT) which catalyzes genome synthesis; and the ribonuclease (H) domain which degrades pregenomic RNA and facilitates replication. HBV X antigen protein (HBxAg) is encoded by ORF-X and has multiple functions that support various stages of viral replication including signal transduction, DNA repair, activation of transcription pathways and inhibition of protein degradation along with participation in the oncogenic potential of HBV [8-11]. In the S-domain, the intermolecular disulfide bonds contribute to the structural stability of spherical virions and promote high resistance of HBV to inactivation by dehydration and heat stress [12,13]. Other important functional elements include direct repeats (DR1 and DR2) required for strand-specific synthesis of DNA during replication and enhancer elements (En1 and En2) which promote liver-specific expression of HBV gene products. Apart from this, a glucocorticoid-responsive element (GRE) sequence within the S-domain, a post-transcriptional regulatory element also exists. This region controls gene transcription and protein activation either by reversible events such as posttranslational modifications of phosphorylation or sequestration; and via irreversible events such as proteolysis. The GRE overlaps En1 and the polyadenylation signal (which makes transcribed RNA more stable, prevents degradation and allows the mature messenger RNA molecule to be exported from the nucleus and translated into a protein by ribosomes in the cytoplasm) within the core gene [14]. To summarize, apart from the major structural proteins, additional functional components in HBV have been demonstrated to enhance replication, promote liver-specific expression of viral proteins, prevent viral protein degradation and improve structural stability during cellular entry.

Updates on Viral Entry

The mode of entry and HBV replication steps within the hepatocyte has been extensively updated in the last decade. The virus attaches to the host cell surface (basolateral membrane of hepatocyte) through initial low-affinity binding on highly sulfated-heparan sulfate proteoglycans (HSPG) such as hepatotropic glypican-5 followed by high-affinity binding on target receptor. This binding to the HSPGs is mediated by electrostatic interactions between the negatively charged HSPG and two positively charged residues of the S-domain [15]. Initial studies showed that heparin, a glycosaminoglycan, interfered with HBV attachment. The higher the sulfation, the stronger the inhibition - lesser sulfated glycosaminoglycans such as chondroitin sulfate were less effective in blocking HBV entry. Thereafter the region between amino acids at position 2 and 47 of the pre-S1 of the HBV acts as receptor binder and attaches to the liver cell entry receptor. The latter was identified as the sodium taurocholate co-transporting peptide (NTCP, coded by the SLC10A1 gene; functions to uptake conjugated bile acids into hepatocytes). It is interesting to note that NTCP expression is rapidly lost after isolation of primary human hepatocytes and is absent in poorly differentiated hepatocellular carcinomas (HCC). Thus, malignant hepatoma cells and primary hepatocytes do not support and are not susceptible to infection with HBV (lack of efficient cell culture system permissive for viral infection and replication). Nonetheless, recently, human pluripotent stem cells transformed to hepatocyte-like cells (HLC) were found capable of expressing hepatocyte markers and host factors needed for the development of HBV infection [16]. Virus internalization into the hepatocyte cytoplasm occurs through the process of endocytosis in which the viral material to be internalized is surrounded by an area of host cell plasma membrane, which then buds off inside the cell to form a vesicle containing the ingested viral material. HBV infection was low in cell lines with overexpressed NTCP. This meant that the co-factors for internalization and infection were important for viral infection. It was identified that the receptor tyrosine kinase, also known as the epidermal growth factor receptor (EGFR), through interaction with the NTCP triggers the internalization and endocytosis process mediated by the caveolae-1/lipid-raft and possibly clathrin, leads to the formation of endosomes in the cytoplasm [17,18]. The host-cell protein, the calcium-dependent cell adhesion E cadherin was shown to play a central role in HBV entry. This protein binds to the glycosylated NTCP and promotes relocation to the basolateral membrane (cell polarization). On a different note, the cell-polarization limits entry of hepatitis C virus through tight junctions that restrict viral access to receptor binding [19].

There are three different types of endosomes: early endosomes, late endosomes, and recycling endosomes, differentiated by their morphology, the time taken for the endocytosed material to reach them, and by markers such as Ras superfamily of G proteins called Rabs. Once endocytic vesicles uncoat, they fuse with early endosomes (via Rab5A) which then mature into late endosomes before fusing with lysosomes (via Rab7A) [20]. In the HBV internalization cycle, the EGFR activation triggers a time-dependent relocalization of HBV pre-S1 to early and late endosomes and to lysosomes in concert with EGFR transport. However, blockade of EGFR-downstream signaling proteins including mitogen-activated protein kinase (MAPK), phosphoinositide 3-kinase (PI3K), and signal transducer and activator of transcription (STAT), does not have a significant effect in reducing HBV infection. Interestingly, efficiency of EGFR endocytosis and HBV entry were reduced when there was a deleterious mutation in EGFR or genetic knockdown of endocytosis adaptor molecules. In this regard, it was demonstrated that the suppression of EGFR ubiquitination by site-directed mutagenesis or knockdown of the EGFR-sorting molecules [signal-transducing adaptor molecule (STAM) and lysosomal protein transmembrane 4β (LAPTM4B)] ameliorated EGFR transport to the late endosome which was shown to be critical for efficient HBV infection. Another novel finding is that the hepatocyte NTCP undergoes extensive oligomerization in the presence of HBV preS1. Oligomerization refers to the interaction of more than one polypeptide chain, which results in the formation of the quaternary structure, generally considered to be the highest level of organization within the protein structural hierarchy. The drug troglitazone (but not pioglitazone) blocked internalization of HBV preS1 and its receptor, NTCP by preventing oligomerization. This work represented the importance of small molecule and peptide-based therapy in prevention of HBV infection [21,22]. Clathrin-mediated virus entry also plays a role in HBV internalization, in which the interaction with protein adapter-2 (AP-2) facilitates infection. Silibinin, a known inhibitor of clathrin-mediated endocytosis was shown to reduce HBV virus entry of HepG2-NTCP cell line [23]. Internalized virus escapes from the endocytic pathway once signaling that support fusion is activated. The crucial aspect in fusion mechanism is dependent on the pH. Some of the identified (but not confirmed) fusogenic domains include the C-terminal half of the pre-S2 region, the N-terminal of the S-region, pre-S1 region and the N-terminal of pre-S1 region [24,25]. To summarize, HBV entry into hepatocytes is not only governed by attachment of pre-S1 to the NTCP receptor, but also initial priming through low-affinity binding with heparan sulfate proteins on hepatocyte, internalization via the tyrosine receptor kinase EGFR, oligomerization to stabilize viral structure orchestrated by multiple other small molecules such as host cell calcium-dependent E cadherin, clathrin and adapter proteins that all form novel drug targets. For example, bafilomycin A1, an inhibitor of vacuolar enzymes responsible for acidification of pH gradient within endosomes inhibited HBV in duck hepatocytes and ameliorated HBV in human cell lines. Further, silencing of small molecules (Rabs) that transport plasma membranes to endosomes also significantly reduced HBV infection. Depending on the structure and biology of HBV, various entry inhibitors have been tested in pre-clinical studies. These include: a. Attachment inhibitors targeting S-,M-,L-HBs (heparin and suramin) or pre-S1 (proanthocyanidins); b. HSPGs (synthetic anti-lipopolysaccharide peptides); c. Substrate inhibitors of NTCP that target NTCP [taurocholic acid and derivatives such as ursodeoxycholic acid (UDCA), tauro-UDCA and glyco-UDCA, irbesartan]; d. Those targeting NTCP + Niemann-Pick C1-Like 1 (NPC1L1) protein (ezetimibe); e. Direct inhibitors of NTCP that interfere (cyclosporine A, vanitaracin A); f. Those that mildly or do not interfere (myrcludex-B, SCY450, SCY995 and Evans blue) with bile acid uptake and; g. Those which directly regulate NTCP expression (Ro41-5253, retinoic acid receptor antagonist) [26,27].

Updates on Nuclear Transport, Assembly and Release

After escape from the late endosome, the viral particles traverse the cytoplasm towards the host cell nucleus. As previously stated, the membrane fusion leads to direct release of nucleocapsids into the cytoplasm. A conserved membrane-permeable peptide within the surface protein of HBV was recently identified, of the pre-S2 domain, called the translocation motif (TLM). The TLM promotes delivery of proteins and nucleic acids into cells and tissues. Surface exposure of TLM peptides on the HBV surface protein due to proteolytic processing leads to fusion of peptides to HBc protein enabling formation of fully assembled capsids [28-32]. These viral capsids then translocate (via microtubule mediated transport) as complete ‘virus’ across cytoplasm towards the nucleus. The microtubule transport assembly is dependent on tubulin distribution and linkage of capsids to the dynein-motor-complex (cytoskeletal motor proteins that move along microtubules). Nocodazole is a drug that can depolymerize microtubules and thus block viral nucleocapsids from reaching the host cell nucleus, preventing formation of covalently closed circular DNA (cccDNA) that defines the HBV life cycle. Nonetheless, microtubule destabilizing drugs are associated with severe side effects and cannot be utilized in clinical setting [33-36]. The viral nucleocapsids undergo disassembly at the host-cell nuclear pore complex where the HBV rcDNA is converted to cccDNA which serves as a template for transcription of viral RNAs (pre-genomic and sub-genomic RNA). HBV pre-genomic RNA contains a stem loop called epsilon which is essential for RNA generation and packaging into viral capsids. It is through interaction with the Zinc finger antiviral protein (ZAP), interferon treatment destabilizes RNA generation and reduces viral replication. Similar to ZAP, recent studies have identified that multiple other small molecules and cellular factors interact with the HBV RNA to promote or suppress degradation and affect viral replication. These include cytidine deaminase, splicing factors, small ribonucleoprotein, RNA-binding motif protein and peroxiredoxins, which also act as small molecular targets for HVB therapy [37-40].

Updates on Viral Transcription and cccDNA

Within the cytoplasm, along with viral polymerase, pre-genomic RNA is encapsidated by HBV core protein to form a viral capsid. Inside the viral capsid, pre-genomic RNA undergoes reverse transcription to generate single-stranded negative-strand DNA (immature nucleocapsids, within infected cells), further followed by generation of partially double-stranded DNA (mature nucleocapsids, in released viral particles) yielding viral rcDNA. These capsids containing rcDNA are either transported back into the nucleus to increase the cccDNA pool or enveloped and released as progeny virions. HBsAg production is predominantly from cccDNA in younger HBeAg-positive patients. Hypo-phosphorylation of capsid proteins produces regular virions while hyper-phosphorylation produces empty virions [41-44]. The reverse transcription also produces aberrant by-products called HBV double-stranded linear DNA that are either released as defective virions or integrate with the host genome. This aberrant integration fails to transcribe pre-genomic RNA (no replicative power), but could still act as a template for generation of HBsAg. This happens in older chronic HBV patients who are HBeAg negative. Currently approved medications for HBV such as interferon-α and nucleos(t)ide reverse transcriptase inhibitors (NAs) reduce viral replication and slow disease progression, but do not cure chronic HBV infection. This is because these agents do not have any effect on persistent HBV cccDNA, since cccDNA formation is not only dependent on the viral DNA polymerase but also the host DNA polymerase(s). In this regard, it was shown that the anti-retroviral host factor SAMHD1 binds to single-stranded virus DNA, acting as a scaffolding protein to facilitate formation of cccDNA through relaxed circular DNA repair processes [45-47]. The HBx protein was demonstrated to activate HBV transcription through recruitment onto cccDNA. HBx also counteracts host restriction mechanisms of cccDNA transcription. Recently, the smallest known proteins with prolyl isomerase activity, which catalyze the cis-trans isomerization of proline peptide bonds, Parvulin 14 and Parvulin 17, were discovered to bind to HBx and cccDNA and promote HBV replication in an HBx-dependent manner. Thus, HBx itself and HBx-involved protein-protein interactions form novel molecular targets for therapeutic development against HBV [48-50]. A recent study found that the Smc5/6 of the structural maintenance of chromosomes family suppresses HBV replication. The drug nitazoxanide was found to block the inhibitor of Smc5/6 (damage specific DNA binding protein 1 or DDB1 binding to HBx protein) and promote suppression of replication [51]. Each infected hepatocyte contains one to 10 cccDNA copies with a half-life of 9.2 months in NA-treated chronic HBV patients. To clear cccDNA from infected cells, apart from direct targeting of cccDNA, two other steps are imperative. First, viral replication and cccDNA replenishment must be completely blocked, and, second, exhaustion of the pool of pre-existing cccDNA within a specified time frame. In the presence of potent suppression of viral replication with an NA addition of small interfering RNA or capsid inhibitor may help clear cccDNA completely [52-54]. 

**Figure 1:**
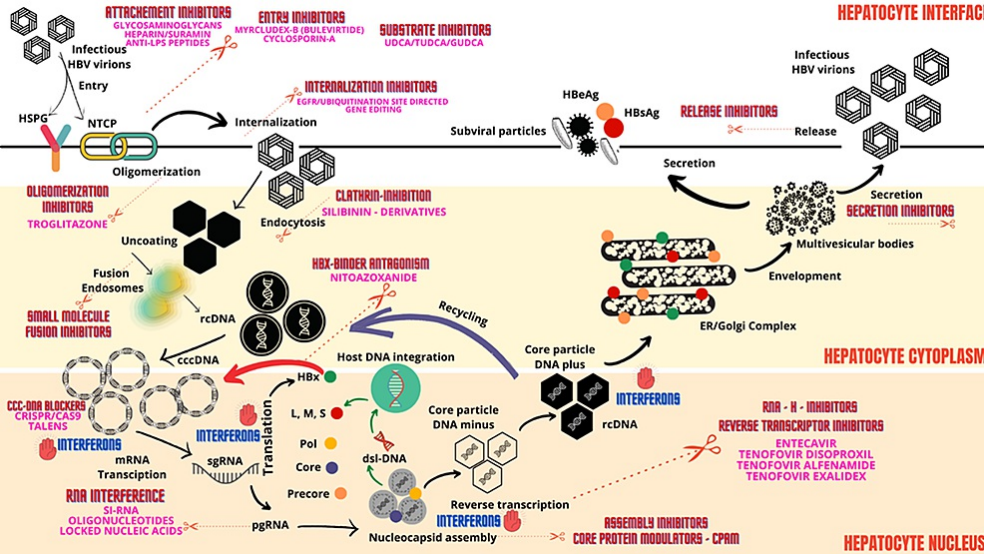
Schematic representation of hepatitis B virus (HBV) life cycle, pertinent steps of host infection and targets for new antiviral therapies. HSPG - heparin sulfate proteoglycans, NTCP – Sodium taurocholate co-transporting polypeptide, LPS – lipopolysaccharide, UDCA - ursodeoxycholic acid, TUDCA – tauro-UDCA, GUDCA – glyco-UDCA, HBeAg – HBV envelope antigen, HBsAg – HBV surface antigen, ER – endoplasmic reticulum, RNA-H – ribonuclease H, rcDNA – relaxed circular DNA, cccDNA – covalently closed circular DNA, mRNA – messenger RNA, siRNA – small interfering RNA, pgRNA – pregenomic RNA, sgRNA – subgenomic RNA, CPAM - core protein allosteric modulator, Pol – polymerase, L – large HBsAg, S – small HBsAg, M – medium HBsAg

With respect to cccDNA clearance, two pathobiological processes are pertinent. One, hepatocyte proliferation itself contributes to reduction in load of cccDNA within infected hepatocytes. In patients with advanced fibrosis or cirrhosis, the hepatocyte replicative senescence adds to the burden of cccDNA formation. Destruction of infected hepatocytes in the presence of potent replication suppression will help reduce cccDNA formation - a therapeutic approach that would require combination of multi-targeted treatment strategies. The cccDNA removal also occurs via non-cytolytic clearance of infected hepatocytes in the presence of antiviral cytokines, specifically interferon-α. It was shown that higher levels of interferon-α were associated with improved cccDNA clearance through triggering of non-cytolytic degradation of cccDNA from infected hepatocytes through induction of the nuclear deaminase A3A or A3B. However, such high doses in a clinical scenario can lead to adverse events and hence are impractical. Recently, the PASylation (addition of polypeptide comprising Proline, Alanine and Serine to increase plasma half-life) of interferon-α was found to improve antiviral effect without additional toxicity [55-59]. Figure 1 summarizes an updated schematic of the HBV life cycle and pertinent therapeutic targets.

Updates on immunopathogenesis, genetic variants and applied biology

Host immune response against HBV infection includes innate immunity and adaptive immunity. The former includes downstream responses that are activated by pattern recognition receptor (PRR), natural killer (NK) cells, NK-T cells, and monocytes and macrophages; while the latter includes cluster of differentiation (CD)4 + T lymphocytes, CD8 + T lymphocytes, and B lymphocytes. In chronic HBV infection, the virus limits and evades antiviral effects of the innate immune and adaptive immune system through various mechanisms, resulting in continuous replication associated with dysfunction of various immune cells. HBeAg is immunomodulatory and is involved in antigen presentation and recognition by CD4+T cells. In HBeAg negative HBV infection (associated with pre-core stop codon mutation), there is rapid progression, cirrhosis and liver cancer development due to amelioration of host innate immune functions. Similarly, the HBV core promoter mutation in enhancer II which results in enhanced viral replication is accompanied by a reduction or loss of HBeAg leading to fulminant or progressive chronic hepatitis. The HBsAg mutant - defect in S region to arginine at amino acid position 145 and loss of group-specific antigenic determinant a (target of vaccine response) - escapes immune surveillance and infection even in the presence of antibodies to surface antigen and also development of occult HBsAg negative HBV infection. Vaccine-escape mutations occurred in particular when lamivudine (currently not utilized) was used in the long term [60-64]. The size of exposure or inoculum determines HBV persistence and clearance. Low-dose inoculum leads to a massive spread of the virus in all of the hepatocytes and viral persistence; whereas high-dose inoculum showed a limited spread of the virus to hepatocytes and rapid viral clearance. This phenomenon depends on the synchronized effector activity of CD4+ and CD8+ T cells. Exhaustion and depletion of CD4+ T cells in limited exposure infection along with synchronized influx of HBV-specific CD8+ T cells in the liver promotes viral persistence. In high viral load, interferons-α/β suppress viral replication through transcriptional and post-transcriptional modification. In early infection and low viral load, HBV utilizes host interferon response to promote viral persistence via stimulation of enhancer I in the genome which interacts with STAT3 and hepatocyte nuclear factor 3γ (HNF3γ). Innate immune activation functions through PRRs recognizing pathogen-associated molecular patterns (PAMPs). These include: a. Toll-like (TLRs; TLR2 activation promotes pro-inflammatory cascade for viral clearance, TLR4 activation through HBsAg related dendritic cell, soluble CD14 dependent cytotoxic T cell mechanism) receptors; b. Retinoic acid-inducible gene I (RIG)-like (dual antiviral effect on pre-genomic RNA through type III interferon induction and HBV polymerase interaction) receptor; c. Nucleotide-binding oligomerization domain-containing protein (NOD)-like, C-type lectin receptors and d. DNA-sensing (cytosolic cGAS recognize HBV DNA, suppress interferon suppressing regulatory factor 3, promote viral persistence) receptors.

Some preclinical studies have shown that, in early HBV infection, PRR-mediated innate immune responses are not activated - the stealth virus phenomenon where the virus interferes with innate signaling pathways to attenuate intrinsic antiviral immune responses [65-67]. HBsAg and HBeAg, in a dose-dependent manner, via interference with c-Jun N-terminal kinases (JNK) activation, inhibits expression of TLR2 mediated IL-12 and tumor necrosis factor-α (TNF-α) production in monocytes and macrophages. HBV also suppressed nuclear factor kappa B (NF-κB), extracellular signal-regulated kinase (ERK)1/2, blocked myeloid differentiation primary response 88 (MYD88) protein expression and inhibited type 1 interferon induction (via HBxAg protein). Recombinant HBx protein-based small interfering RNA (siRNA, short interfering RNA or silencing RNA) recovered interferon-1 activity by activating RIG-1 pathway. Nonetheless, detailed molecular determinants for potential recognition of HBV PAMPs by PRR still remain to be elucidated. This would increase the therapeutic armamentarium to include PRR agonists that would help in viral clearance [68-70]. 

Cellular Level Immune Activity in HBV Infection

NK cell dysfunction is also central to viral persistence in HBV infection. The ability of myeloid DCs to activate NK cells is impaired due to weak action in decreasing activating cytokines (IL-6, IL-12, IL-18) resulting in reduced secretion of interferon-γ and lowered activity of interferon-α. IL-10 secretion from Kupffer cells (liver resident macrophages) promotes cytokine blunting and hence lowers NK activation. IL-10 is an immune-suppressive cytokine (also secreted by virus-specific B lymphocytes) that maintains the immune tolerance during persistent HBV infection. Programmed death-ligand 1 (PD-L1) on suppressive monocytes also inhibit autologous NK cell activation. Reduction in the NK-cell mediated cytotoxic prowess and IFN-γ production contribute to HBV persistence. The expression of activating receptors on NK cells such as the NKG2D and 2B4 are also reduced in chronic HBV infection.

The myeloid-derived suppressor cell (MDSC) with predominant granulocytic subset (gMDSC) and monocytic MDSC (mMDCS) has an inverse relation with T cell function and hepatitis in chronic HBV infection. MDSCs potentiate CD4+ and CD8+ T cell responses via arginase-dependent pathways. High arginase levels reduce amount of arginine required for lymphocyte physiology and growth and resulting in lymphocyte dysfunction. Disruption of MDSC differentiation and T-regulatory cells (Tregs) resulted in immunosuppressive cytokine reduction which inhibited HBV replication. NKT cells of a special subset of T lymphocytes that express surface markers of T lymphocytes and NK cells - the invariant NKT cells (iNKT) lose functionality in the presence of HBV infection through an increase in T-cell immunoglobulin and mucin domain-3 (Tim-3) and programmed cell death protein 1 (PD-1) - antiviral treatment or Tim-3 blocking restores immune function of iNKT cells and improves viral clearance [71-74].

Interferon-γ secreted by lymphocytes in HBV infection induces Kupffer cells to produce chemokine (C-X-C motif) ligand 9 (CXCL9) and recruits HBV-specific CD4 + T lymphocytes to enter the liver for apoptosis, leading to chronic HBV. Defects in CD8 + T-lymphocyte functions through multiple pathways [blunted cytokine responses, T-lymphocyte depletion, high expression of co-inhibitory molecules such as Tim-3, PD-1 and CTLA-4, upregulation of TNF-related apoptosis-inducing ligand (TRAIL), arginase secretion] result in reduced HBV clearance from hepatocytes. PD-1 blockade can partially restore B cell function and CD 8+ T cell functions for viral clearance. Higher level of T helper cell 17 (Th17) lymphocytes (secretes IL-17, IL-21, IL-22) in the liver and peripheral blood was associated with acute and acute on chronic liver failure due to HBV. In chronic HBV infection, a follicular helper T (Tfh) cells response to HBsAg was required for HBV clearance which was blocked by Treg cells [75-82].

**Figure 2:**
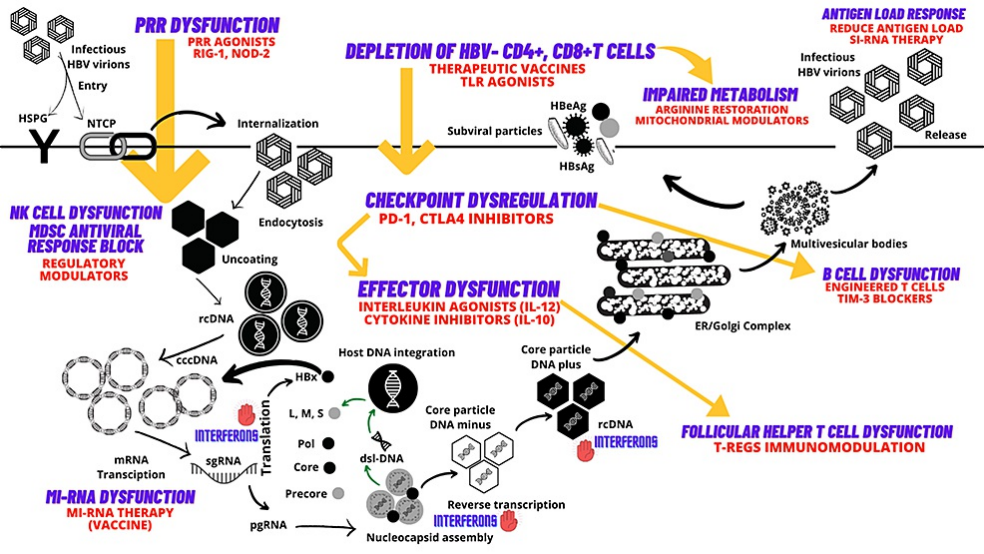
Schematic representation of hepatitis B virus (HBV) immuno-pathogenesis and immune targets of new antiviral therapies. HSPG - heparin sulfate proteoglycans, NTCP – Sodium taurocholate co-transporting polypeptide, TLR – toll-like receptors, CD – cluster of differentiation, PRR – pattern recognition receptors, PD - programmed cell death protein, CTLA - cytotoxic T-lymphocyte-associated protein 4, Tregs – T-regulatory cells, IL – interleukins, TIM-3 - T cell immunoglobulin and mucin domain-containing protein 3, miRNA – micro-RNA, NK – natural killer cells, MDSC - myeloid-derived suppressor cell, HBeAg – HBV envelope antigen, HBsAg – HBV surface antigen, ER – endoplasmic reticulum, RNA-H – ribonuclease H, rcDNA – relaxed circular DNA, cccDNA – covalently closed circular DNA, mRNA – messenger RNA, siRNA – small interfering RNA, pgRNA – pregenomic RNA, sgRNA – subgenomic RNA, CPAM - core protein allosteric modulator, Pol – polymerase, L – large HBsAg, S – small HBsAg, M – medium HBsAg

Endoplasmic reticulum (ER) stress also plays an important role in viral persistence. In HBV-infected cells, a large number of viral surface proteins are folded in ER during the replicative phase, resulting in disruption of ER homeostasis and ER stress. This is identified as ground-glass hepatocytes that accumulate ER mutant surface proteins (pre-s1 and pre-s2 mutants) which represent ER hypertrophy. ER stress leads to activation of ER degradation enhancers and hence reduction in the immune responses for viral clearance. The intracellular imbalance in favor of L-HBs compared with M- and S-HBs leads to ER stress, which can trigger cellular signals for apoptosis or uncontrolled cellular growth [83]. HBV RNA directly degrades host micro-RNA (miRNA, which is non-coding) leading to reduction in levels of miRNA-122 [block fibrosis by blocking collagen synthesis via transforming growth factor beta (TGF-β) pathway], miRNA-15 family and miRNA-let-7 family which lead to increased HBV replication, liver fibrosis and carcinogenesis [84-90]. Based on our current understanding of HBV immunopathogenesis, novel treatment strategies for enhancing chances for clinical cure of chronic HBV infection include PRR, TLR7 or RIG-I agonists (increases innate immune responses), PD-1 blockade (immune checkpoint blockers), therapeutic vaccines (based on miRNA), and chimeric antigen receptor T lymphocytes that improve adaptive immune responses for enhancing viral clearance. Figure 2 summarizes an updated schematic of the pertinent immunopathogenic processes and therapeutic targets in HBV infection.

Updates on HBV genotypes and their clinical importance

Currently, 10 genotypes of HBV exist, with additional subtypes (mutations or recombinant strains), which are identified by the letters A to J and numbered respectively, which, through genetic mutations and the lack of proofreading in reverse transcriptase, have evolved over the long term, creating challenges to their elimination. An example is the HBV genotype B2 which is a recombinant, with majority of the genetic framework from HBV genotype B, and the precore/core region from genotype C. Coinfection with different HBV genotypes and intergenotypic recombination of HBV strains are extensively documented. Most commonly associated recombinants include genotypes B/C or A/D. Each genotype is classified by an 8% or more divergence in the nucleotide sequence of the genome. Genotypes A to D are the four predominant genotypes; B and C are most common in eastern and southeastern Asia, A and D are found in North America, Africa and Europe and genotype E is found in West Africa. Genotypes A and B have a greater response to interferon therapy than C and D, but none of the genotypes have differential responses to oral antivirals [91-93]. Delayed HBeAg seroconversion and a higher risk of reactivation in the HBeAg-negative phase were notable in HBV patients with genotype C who also have more advanced fibrosis. Liver cancer develops in young patients without cirrhosis who harbor HBV genotype B-related infection. Patients with HBV genotypes C and D, compared with genotypes A and B, have late or absent HBeAg seroconversion after multiple hepatitis flares that accelerate progression of liver disease, conferring worse clinical outcome. HBV genotypes are also associated with specific virological manifestations such as higher frequency of basal-core promoter A1762T/G1764A variants, pre-S deletion mutations, greater viral replicative burden, expression of intracellular HBV DNA and core protein expression and HBeAg secretion in genotype C when compared with other genotypes. In a systematic review and metanalysis, authors found that the blood group B was associated with a lower risk of HBV infection and persons with blood group O had a 12% increased risk of HBV infection in endemic regions [94-97].

Evaluation and treatment of HBV related liver disease

Current Approaches to Diagnosis and Evaluation

Presence of HBsAg indicates acute or chronic infection and is the first serologic marker to appear. HBV infection is considered chronic if HBsAg persists beyond six months. The HBeAg indicates active replication while its absence can also indicate mutations in the pre-core region of the e-antigen that prevent production of HBeAg. Antibody response to HBeAg (anti-HBe) indicates that the virus is non-replicative, but is also seen among HBV patients with HBeAg mutation with active disease. Antibody to HBc antigen can be present in acute infection and reactivation (IgM) and with past exposure to HBV (IgG). It can be seen in solitude when antibody response to HBsAg is waning. Patients who are HBsAg-positive and anti-HBc-positive need further evaluation for initiation of treatment. Those who are anti-HBs-positive and anti-HBc-positive are considered to have infection in the past and currently resolved. Nonetheless, in these patients infection may remain latent, only to reactivate under special circumstances (immunosuppression, spontaneous mutation) along with re-emergence of HBsAg. Patients who are HBsAg, anti-HBs, and anti-HBc negative do not possess immunity needs vaccination. Patients who are only anti-HBs-positive are immune or have undergone vaccination [98-100].

In patients who are HBsAg and antibody (total) to HBcAg positive, further differentiation is made on the presence or absence of HBeAg after which classification into chronic infection and chronic hepatitis is made for treatment decisions. Patients with chronic infection have normal alanine aminotransferase (ALT) and no or minimal liver damage (fibrosis grade <2) while those with chronic hepatitis have elevated HBV DNA and ALT with ongoing necro-inflammatory liver damage or significant fibrosis (grade ≥2), for whom treatments are to be directed [101,102]. The alanine transaminase concentrations generally correlate with hepatic necroinflammation in HBV patients. High-normal ALT levels ranging from 40 to 70 IU/liter are linked to cirrhosis and liver-related deaths. Currently, guidance on recommendations suggests that the ALT cutoffs should be 35 U/liter for males and 25 U/liter for females and significant elevation is considered two times the upper limit of normal (ULN). Even though percutaneous liver biopsy and histological interpretation is the gold standard for fibrosis assessment, its use in clinical practice is limited and follow-up biopsies are not routinely employed due to patient unacceptance. In this regard, assessment of hepatic fibrosis by non-invasive modalities is suggested. These include shear wave elastography (transient, acoustic radiation force impulse, or multidimensional) as well as magnetic resonance elastography (MRE). Among these the FibroScan® transient elastography is the best validated worldwide. In obese patients and those with ascites, MRE is to assess liver stiffness measurement (LSM) with phase contrast imaging is suggested, which can also stage even mild fibrosis, but it is less cost effective, not well tolerated and more time consuming than ultrasound methods. Other validated serum biomarker combinations for diagnosis of significant fibrosis in HBV patients include the aspartate aminotransferase (AST)-to-platelet ratio index (APRI, low sensitivity in African patients), the Forns index, Fibrotest®, Fibrosure™, Fibrometer® and enhanced liver fibrosis (ELF™) score [103-106].

There are three types of treatment end points or cure in HBV infection. In functional cure, there is resolution of clinical infection which is sustained off drug treatment - no inflammation, normal ALT level and normal liver biopsy, HBsAg seroclearance (equal to 0.05 IU/ml in serum) with or without the emergence of anti-HBs. Protective immunity is when the anti-HBs level is greater than 10 IU/ml. Complete cure is virologic cure, consisting of all the elements of functional cure plus loss of cccDNA within the liver. In clinical practice, most of the treated patients fall into an interim cure period in which there is disease inactivity - absence of inflammation (normal ALT level and liver biopsy), low or undetectable HBV DNA level in the presence of HBsAg positivity. In this situation, a patient with chronic hepatitis is effectively down staged to one with chronic infection. An HBsAg level of 100 IU/ml in Asian HBeAg-negative patients is predictive of spontaneous HBsAg seroclearance within six to eight years. Inactive - low replicative chronic HBV patients have high rates of spontaneous HBsAg seroclearance, - 8.1% and 44.7% after 10 and 25 years of follow-up, respectively [107-110]. In a nutshell, HBsAg patients who require treatment with antiviral agents include patients with chronic hepatitis, those with cirrhosis (any level ALT, detectable HBV DNA and decompensated patients irrespective of DNA and ALT levels), those with hepatocellular carcinoma (HCC), HIV coinfection, on chemotherapy or biologic and immunomodulatory agents, women in the third trimester of pregnancy if HBV DNA is greater than 200,000 IU/mL and those with extrahepatic manifestations such as glomerulonephritis and vasculitis [111,112]. A summary of various international guidelines for the management of chronic HBV is shown in Table 1.

**Table 1:** Summary of guidelines for treatment of chronic hepatitis B virus infection WHO – World Health Organization, ATA – American Treatment Association, APASL Asia-Pacific Association for the Study of Liver, EASL – European Association for the Study of Liver, AASLD – American Association for the Study of Liver Diseases, APRI -  aspartate transaminase (AST) to Platelet Ratio Index, HBV – hepatitis B virus, ALT – alanine aminotransferase, ULN – upper limit of normal, ETV – entecavir, TDF – tenofovir disoproxil, TAF – tenofovir alafenamide, IFN – interferon, LAM – lamivudine, ADV – adefovir, LdT – telbivudine

Management	WHO 2015	ATA 2015	APASL 2015	EASL 2017	AASLD 2018
When to initiate	Compensated or decompensated cirrhosis (or APRI > 2 in adults) Age >30 yr, persistently abnormal ALT, and HBV DNA > 20,000 IU/ml HBV DNA not available, then on bases of persistently abnormal ALT levels	ALT level >2 x ULN and HBV DNA >2,000 IU/ml Compensated/decompensated cirrhosis with detectable HBV DNA	ALT >2 x ULN and HBV DNA >2,000 IU/ml in HBeAg negative or > 2,000 IU/ml in HBeAg-positive Compensated or decompensated cirrhosis with detectable HBV DNA	HBV DNA >2,000 IU/ml, ALT >ULN and moderate liver necro-inflammation or fibrosis (F2 minimum) Compensated or decompensated cirrhosis with detectable HBV DNA HBV DNA >20,000 IU/ml and ALT ≥2 x ULN HBeAg positive, high HBV DNA level, and age 30 yr Family history of HCC, cirrhosis, extrahepatic manifestations	ALT >2 x ULN and HBV DNA >2,000 IU/ml in HBeAg negative or > 20,000 IU/ml in HBeAg positive Cirrhosis with HBV DNA > 2,000 IU/ml Age 40 yr, family history of HCC, previous treatment, extrahepatic disease
What to treat with	ETV, TDF	ETV, TDF, Peg IFN-α	ETV, TDF, Peg IFN-α or LAM ADV and LdT (less preferred)	ETV, TDF, TAF, Peg IFN-α	ETV, TDF, Peg IFN-α
When to stop	Lifelong treatment in cirrhosis Stop treatment if non-cirrhotic, HBeAg seroconversion, or persistently normal ALT levels with or without undetectable HBV DNA Stop treatment in case of persistent HBsAg loss with 1 yr of consolidation therapy	HBsAg loss for 6–12 mo	HBsAg loss for at least 12 mo Non-cirrhotic HBeAg seroconversion and undetectable HBV DNA after minimum 1 yr (preferably 3 yr) of consolidation therapy Non-cirrhotic HBeAg negative and undetectable HBV DNA for ≥2 yr	HBsAg loss Non-cirrhotic HBeAg positive with seroconversion and undetectable HBV DNA after 12 mo of consolidation therapy Noncirrhotic HBeAg negative with undetectable HBV DNA for 3 yr	HBsAg loss Lifelong in cirrhosis
Retreatment	Reactivation of HBV	Relapse of HBV with respect to HBV DNA and ALT levels (specific levels not provided)	None	Similar to treatment-naive patients	None

It is important to note that among HBsAg-positive cases who additionally suffer from obesity or metabolic syndrome, the risk of development of cirrhosis is higher than those with HBV infection alone [111,112]. In these patients, if HBV DNA level is low or undetectable then the abnormal ALT level may be due to non-alcoholic fatty liver disease (NAFLD) which requires targeted treatments and lifestyle modifications with close follow up. However, in those with increasing ALT, only a liver biopsy can help differentiate between NAFLD-related liver disease or HBV-associated necro-inflammation. Current literature suggests that patients in the immune tolerance phase or HBeAg positive chronic infection (very high HBV DNA >10^7^ IU/mL and normal ALT) if aged above 30-40 years benefit from antiviral therapy irrespective of other standard inclusions for treatment initiation. Nonetheless, one must be aware that spontaneous HBeAg and HBsAg clearance with remission of liver disease can occur in 70 - 80% of patients at median follow up of approximately 10 to 20 years [113-115].

New updates on diagnosis and monitoring

The HBV core-related antigen (HBcrAg) is a new indicator that encompasses amino acid sequence common to HBeAg and HBcAg as well as the 22-kDa precore protein. HBcrAg positivity correlates with intrahepatic HBV DNA and pregenomic RNA levels among patients on antiviral treatment. This makes HBcrAg measurement a good serum marker of the active transcriptional activity of liver cccDNA and higher levels correlate with increased risk of hepatocellular carcinoma [116-118]. Another potentially new viral marker for future clinical use is measurement of HBV RNA which has been shown to provide significant insights into antiviral treatment response and cessation decision; identification of functional cure in chronic HBV infection; risk for HBV-related liver cancer and levels of intrahepatic HBV cccDNA. Particularly, the HBV pre-genomic RNA reflects viral replication activity and could be a very valuable tool for monitoring the effect in patients receiving novel anti-HBV therapies. These viral molecules are promising as surrogate markers for HBV viral activity, and when used alongside standard biomarkers, they allow for better assessment of HBV infection and treatment responses [119-121]. Since carcinogenesis is an important aspect in the natural history of HBV-related infection, the novel HBV DNA quantitation-time index (HDQTI), comprising HBV DNA quantitation and follow up, was found to predict HBV associated liver cancer prognosis identified a cut-off value at 34. The HDQTI also predicted cancer recurrence and the need for shorter surveillance intervals with appropriate imaging in patients with a high score [122]. Liver biopsy can be avoided in a significant number of patients with use of the combined ELF™ (based on Fibroscan®) algorithm. The optimal cut‐off values of ELF™ were 8.4 to exclude advanced fibrosis, and 10.8 to confirm advanced fibrosis and LSM ≤6.0 kPa and ≤7.5 kPa excludes ≥F3 fibrosis while LSM>9.0 kPa and >12.0 kPa diagnose ≥F3 fibrosis in normal and elevated (1-5× ULN) ALT, respectively [123].

Streamlining the HBV diagnostic process to identify those who would benefit from screening, surveillance or therapy through artificial intelligence-related machine learning (ML) is a novel technique. With the help of ML, a predictive model for inflammation grades of chronic HBV was proposed utilizing a combination of gene expression data and three clinical parameters (ALT, AST, HBV DNA) over which a user-friendly web tool (LiveBoost™) was applied for the clinical prediction of hepatic fibrosis. It was demonstrated that the ML system outperformed FIB-4 scoring in predicting advanced hepatic fibrosis. Additionally, an artificial neural network (ANN) model was found to be effective in diagnosing liver fibrosis regression in HBV patients on therapy. ML-based models were also found to accurately identify persons at risk for HBsAg positivity, predict HBsAg seroclearance, predict treatment decisions in HBV carriers; 28- and 90-day mortality of HBV related ACLF, determine viral variants; and interactions between viral and host proteins to map pathways in hepatocellular carcinoma [124-128].

Current measures and updates on prevention and treatment

The immunogenic first-generation active HBV vaccines were made from materials extracted directly from plasma of chronically HBV-infected patients. It was not the virions that were utilized, but the large amounts of non-infectious spherical viral particles in carrier plasma, which were easily separated by biophysical methods. After cloning the HBV genome, large-scale production of spherical viral particles within recombinant yeast cells (yeast-derived second-generation vaccines) showed comparable protection with plasma-derived vaccines. Vaccine response rates with yeast-derived vaccines are>99% among infants and adolescents, but insufficient in 5% of healthy adults. The majority of yeast-derived vaccines consist only of the S-HBs of the globally underrepresented HBV genotype A2, dominant only in Northern Europe and North America. Very high anti-HBs titers (> 1,000 IU/L) are protective, but low or waning anti-HBs-titers over time increase risk of breakthrough infection with antigenically distant HBV genotypes (HBV genotypes B, C, D and F and at anti-HBs titers of less than 100 IU/L). The minimal infectious dose of HBV is as low as 16 virions (or 3 IU) when transmission occurs through HBV-contaminated blood transfusions [129-132].

Current therapies for the management of HBV include interferon-α (standard or pegylated) and orally administered NAs. First-line therapy should be with an oral antiviral with a strong genetic barrier to viral resistance such as either entecavir, tenofovir disoproxil (TDF) or tenofovir alfenamide (TAF, prodrug of TDF with more stable concentration in serum hence lower dose and less systemic exposure). Short-term treatment with NA is feasible in HBeAg-positive patients experiencing seroconversion to anti-HBe during treatment. A randomized controlled study (FINITE) analysed outcome when TDF therapy was withdrawn in a set of HBeAg negative patients who had achieved suppression of HBV DNA. Interestingly, 43% of patients achieved either HBsAg loss or suppressed DNA without any significant safety concerns [133-136].

Peginterferon for chronic HBV-related hepatitis is not widely used, even though the treatment period is finite (48 wks therapy). For HBeAg positive patients with low HBV DNA (<2 × 10^8^ IU/mL), genotype A, and elevated serum ALT (> 2-5 times ULN) along with necroinflammation on liver biopsy, peginterferon-α could be used as first-line antiviral agent. HBeAg negative, genotype D patients who do not experience decrease in HBsAg levels and 2 log10 IU/ml reduction of HBV DNA at 12 weeks peginterferon-α treatment are considered non-responders. The HBsAg level is useful for prediction and motoring of response to therapy with peginterferon. HBeAg-positive patients, with HBsAg level of 20,000 IU/ml at week 24 are considered non-responders and treatment can be stopped early. Peginterferon leads to higher rates of HBeAg and HBsAg loss at one year mainly in patients with genotype A infection. Overall rates of sustained response (HBeAg seroconversion and undetectable HBV DNA in HBeAg positive patients and DNA <2000 IU/mL in HBeAg negative patients) after a one-year course of treatment is 27-36% and 28% respectively. Combination of NA and peginterferon can be performed via two protocols - de novo combination or the simultaneous administration of the two agents in treatment-naïve HBV patients; and the sequential combination, which features “add-on” or “switch-to” strategy in those who are already on treatment with either drug. This strategy improves HBsAg loss. Nonetheless, the benefits are mainly limited to specific group of patients - those with low baseline HBsAg level and on-treatment HBsAg response, high baseline ALT and viral load and genotype A. Peginterferon should not be used in decompensated cirrhosis, but can be used with caution in patients with compensated cirrhosis [137-139].

For those with chronic HBV hepatitis and multiple drug-resistant virus strains, combination of TDF and entecavir seems to be an effective and safe rescue option. In general, after HBeAg seroconversion, the treatment should continue for at least one year and possibly an additional three years to achieve long-lasting response once therapy is discontinued [137,139]. This three-year continuation phase lowers relapse rates to <30% and hastens loss of HBsAg. Nonetheless, higher relapse rates after NA discontinuation occur in older patients and those with HBV genotype C infection. Ideally, NAs can be withdrawn in HBeAg negative patients only after confirmed loss of HBsAg, with or without antibody development. Recommendations from European and Asian countries suggest stopping of NAs in HBeAg-negative patients who have undetectable HBV DNA at three different times points, six months apart [139,140]. One should not stop NA in patients with cirrhosis. Long-term NA therapy can decrease the cccDNA pool of infected hepatocytes through inhibition of nucleocapsid recycling but cannot prevent the initial cccDNA formation in newly infected hepatocytes [140,141].

In patients with liver failure, benefits were observed in those with model for end-stage liver disease (MELD) score between 20-30, while the mortality rate in those with MELD >30 was >90% even in the presence of early antiviral treatment - these patients need early referral for liver transplantation [141,142]. After liver transplantation, antiviral therapy is indefinite, regardless of HBsAg, HBeAg, or HBV DNA status. For patients on immunosuppressive therapy, antiviral therapy should be continued for at least six to 12 and 12 to 18 months after completion of therapy, as per American and European guidelines respectively; longer duration specifically in those receiving rituximab [142,143]. For pregnant HBV patients, antiviral therapy should commence at 28 weeks gestation and continued 12 weeks post-partum. For patients with HCV and HBV co-infection, entecavir has the least drug-drug interaction and treatment can be started simultaneously. In people living with HIV and HBV co-infection, the treatment should include either TDF or TAF + lamivudine or emtricitabine along with other HIV drugs [142-144].

Future directions for HBV treatment

HBV entry inhibitors targeting NTCP receptors include myrcludex-B (also called bulevirtide, subcutaneous route) and cyclosporine A (CsA). The former, a synthetic lipopeptide derived from pre-S1 domain blocks infection of new hepatocytes and hinders amplification of intrahepatic cccDNA of infected hepatocytes. The latter, a cyclic non-ribosomal peptide inhibit NTCP transporter activity blocking viral entry into hepatocytes - but can impair sodium dependent bile acid uptake resulting in various adverse events. Nonetheless, recently discovered SCY450 and SCY995 derivatives of CsA do not impair bile acid uptake [145,146].

APOBEC3 cytidine deaminase activators (through lymphotoxin-β receptor, LTBR pathway) via engineered non-lytic T cells with HBV-specific T-cell receptors inhibited HBV replication in small animal models. LTBR agonists were found to degrade cccDNA and exhaust intrahepatic pool. Genome-editing using transcription activator-like effector nucleases (TALENs), and the clustered regularly interspaced short palindromic repeats/Cas9 (CRISPR/Cas9) or locked nucleic acid technology (LNA) can be used to target specific DNA sequences for cleaving. TALENs comprise a nonspecific Fok1 nuclease domain fused to a customizable (can be engineered to target and disrupt any specific DNA sequence) sequence-specific DNA-binding domain. However, the safety of such DNA sequence cleavage on ‘HBV integrated host genome’ and its consequences remain to be studied. HBV-specific CRISPR/Cas system-mediated removal of the full-length integrated HBV DNA and the disruption of HBV cccDNA in a stable HBV cell line was demonstrated recently. CRISPR/Cas9 system from *Streptococcus pyogenes* and *S. thermophilus* targeting conserved regions of the HBV genome resulted in degradation of > 90% HBV cccDNA by six days. Nonetheless, even though deep sequencing revealed that Streptococcus-CRISPR/Cas9 had no effect on the host genome, it induced intrinsic off-target adverse effects such as mutagenesis [147-150].

RNA interference (RNAi) by which siRNA produces gene silencing at the post-transcriptional level to downregulate the expression of targeted genes is another novel therapeutic area. The siRNA therapeutic, ARC-520 that targets cccDNA-derived pre-genomic RNA was found to reduce HBsAg levels in HBeAg-positive patients, but not HBeAg-negative patients as in the latter, HBsAg arises not from cccDNA pool, but from HBV DNA integrated with the host genome. The novel ARO-HBV (JNJ-3839), targeting two sources of HBsAg, pre-genomic and integrated DNA, is currently under evaluation. Another siRNA molecule called AB-729 using novel conjugated N-acetyl galactosamine delivery technology with strong anti-HBV activity which acts on all HBV RNA transcriptions is under evaluation [151-153].

Virus nucleocapsid assembly inhibitors/modulators (heteroaryldihydropyrimidines that function to misdirect formation of aberrant or non-capsid structures; and phenylpropenamides or sulfamoylbenzamides that function to produce dysfunctional intact empty capsids) limit HBV replication by causing capsid destabilization is under multiple trials. A novel acyclic nucleotide phosphonate called besifovir is recently approved for trial studies. The main adverse event noted was L-carnitine depletion (myonecrosis, hypoglycemia) in treated patients requiring supplementation. Another new lipid conjugated nucleoside analogue under clinical development is tenofovir exalidex (TLX) which shows enhanced hepatic targeting that maximizes liver activity while reducing systemic drug exposure and was found to enhance HBsAg loss and reduce the cccDNA amount. Another novel nucleoside analogue under phase II clinical study is CMX157 [154-157]. Inhibitors of ribonuclease H (α-hydroxytropolones, N-hydroxyisoquinolinediones and N-hydroxypyridinediones) limit degradation of HBV pre-genomic RNA during DNA minus strand synthesis thereby permitting plus-strand synthesis are a novel class of antiviral agents that block release of infectious virions and amplification/replenishment of cccDNA pool. Inhibiting HBsAg release ameliorates T cell tolerance, reduces T cell exhaustion and restores HBV-specific T cell-mediated immune response. Phosphorothioated oligonucleotide assembly blockers are nucleic acid polymers that prevent assembly of subviral particles which are the primary source of circulating HBsAg. Designated REP 301 and REP 401, these drugs used along with NAs or peginterferon may have better chances at promoting functional cure [158-162].

Based on our advances in understanding immunopathogenesis of HBV, multiple immune modulating therapeutic agents are under development which would help promote functional cure of HBV. TLR agonists (TLR-7 - oral vesatolimod or selgantolimod and TLR-8) induce endogenous interferon production, activate innate responses, leading to induction of interferon-stimulated genes (also called STING agonists) and other signaling cascades that inhibit HBV replication. Nonetheless, phase II studies have shown that even though T cell increase, NK cell responses and interferon signaling were improved with TLR agonists, reduction in HBsAg levels were not identified. This means that monotherapy with these agents is probably of low clinical relevance and hence combination strategies are warranted. Pattern recognition receptor agonists such as RIG-I and NOD-2 agonists activate interferon signaling pathways and proinflammatory cytokines that improve viral clearance. The RIG-I agonist, inarigivir soproxil, a novel oral modulator of innate immunity when used along with TDF significantly increased reduction of HBV replication, HBV RNA and HBsAg levels in a dose-dependent manner in both HBeAg-positive and HBeAg-negative patients [163-165].

PD-1, a highly expressed inhibitory receptor on HBV-specific T cells, along with increased expression of PD-L1 (PD-1 ligand), contributes to T cell exhaustion and high HBV replication in chronic HBV. Thus, PD-1/PD-L1 pathway blockers induce proliferation of HBV-specific T cells, thus restoring functioning T cells and helping control HBV. A pilot study showed that the PD-1 blocker nivolumab along with HBV therapeutic vaccine GS-4774 achieved significant and sustained HBsAg loss [166-168]. Finally, the novel therapeutic protein-based vaccines that include subunit vaccines (HBsAg+HBcAg called HeberNasvac) and antigen-antibody complex vaccines (HBsAg+HBV immunoglobulin) did not demonstrate favorable results due to non-induction of cytotoxic T cell responses. DNA-based vaccines encoding HBV envelope proteins such as INO-1800 (multi-antigen vaccine encoding HBsAg and consensus HBcAg sequence) that induce HBV-specific T cells; INO-9112 (encoding human IL-12); HB-110 (encoding HBsAg, pre-S1 Ag, HBcAg, HBV polymerase, human IL-12) are under evaluation. New vector-based vaccine GS-4774, a recombinant, heat-killed, *Saccharomyces cerevisiae* yeast-based vaccine expressing HBsAg, HBcAg, and HBx, did not provide significant reductions in serum HBsAg levels when used alone, but induced strong immunomodulatory effects when used along with TDF. The non-replicative adenovirus 5 vector vaccine TG1050 encodes a large fusion protein made of modified HBV core, HBV polymerase, and selected envelope protein domains. TG1050 was found to have a good safety profile and induced appreciable HBV-specific cellular immune response in early trials [169-171].

In another study, 12 chemical compound candidates for alpha-glucosidase inhibitors were identified from a library of chemical compounds and used to treat fresh human hepatocytes infected with HBV and monitored for their anti-viral effects. It was found that HBV replication was inhibited by one candidate, a tetramethylpiperidinol derivative in a dose-dependent manner, through interaction with HBV nuclear transcription factor Sp1 which was also associated with significant reduction of cccDNA production, compared to entecavir [172]. To summarize (Table 2), novel therapeutic agents targeting functional cure for chronic HBV infection include entry inhibitors, cccDNA disruptors, translation inhibitors, capsid assembly blockers, polymerase and secretion inhibitors and state-of-the-art therapeutic vaccines [173-176].

**Table 2:** Summary of novel antiviral therapies for chronic HBV infection HBV – hepatitis B virus, ccc- covalently closed circular, Ca – calcium, Mg – magnesium, TALENs - transcription activator-like effector nucleases, CRISPR-Cas9 - CRISPR-associated protein 9, TLRs – Toll-like receptors, PD – programmed death cell receptor, MVA - Modified Vaccinia Ankara, E – intramuscular – electroporation and intramuscular

Type of drug	Name of drug	Clinical trial phase	Route	Mode of action
Entry inhibitors	Myrcludex-B / Bulevirtide	II	Subcutaneous	HBV entry blockade
Oligonucleotides	INOIS-HBVRx (GSK3228836), INOIS-HBVLRx (GSK33389404)	II, Preclinical	Subcutaneous	Antisense Antisense
Core protein allosteric modulators (CpAMs)	RO7049389 JNJ, 56136379 JNJ, 64530440 AB-506, ABI-H2158, ABI-H0731 (Vebicorvir), GLS4JHS, NVR 3–778 QL-007	I to II	All oral	Core protein binding Assembly modulation Assembly modulation Core protein binding Core protein binding Core protein binding Core protein binding Assembly modulation Assembly modulation
HBx protein inhibitors	Nitazoxanide, CRV-431	II, I	Oral Oral	cccDNA transcription Cyclophilin inhibitor
RNA interference	GSK3389404, ARO-HBV (JNJ-3989), AB-729, ALN-HBV (VIR-2218), ARC-520, DCR-HBVS	II to II	Subcutaneous or Intravenous Subcutaneous	RNA degradation RNA interference RNA interference RNA degradation RNA interference RNA interference
HBsAg release inhibitors	Nucleic acid polymers REP 2139 - Ca, REP 2165 - Mg	II	Intravenous Intravenous	Binding and prevents release of HBsAg surface protein
HBsAg neutralization	GC 1102 (Lenvervimab)	II	Intravenous	Neutralization and inhibiting reentry
Inhibitors of cccDNA	TALENs CRISPR-Cas9	Preclinical	Unknown	cccDNA disruption cccDNA disruption
Cell intrinsic and innate immune responses (Toll-like receptor agonists)	RO7020531 Vesatolimod, GS-9620 Selgantolimod, GS-9688 AIC649	I to II	Oral, Oral, Oral	TLR-7 agonist TLR-7 agonist TLR-8 agonist TLR-9 agonist
Immune checkpoint inhibitors	Nivolumab , Cemiplimab	I, I/II	Intravenous	PD-1 blockade PD-1 blockade
Therapeutic vaccines	TG1050/T101, INO-1800, ChAdOx1 HBV, Hep-Tcell, JNJ-64300535, GS-4774	I to II	Subcutaneous or intramuscular	HBV proteins DNA plasmid Adjuvanted ChAd+MVA vector HBV peptide+ TLR9 adjuvant IC31 Electroporation DNA vaccine DNA vaccine

Updates on HBV-related ACLF

Acute on chronic liver failure is a recently described entity in the natural history of cirrhosis, defined by acute insult leading to rapid hepatic decompensation, multiple organ failure and a high risk of short-term mortality, usually less than four weeks. Acute alcoholic hepatitis, drug-induced liver injury, infections and surgical stress are the most frequent precipitants for ACLF. Central to the pathophysiology of ACLF is the state of unchecked persistent inflammation and immune dysfunction with increased propensity to sepsis and organ failure. Reactivation of chronic HBV infection is an important and modifiable cause for ACLF [177,178]. Studies have reported an approximately 35% incidence of ACLF in patients with underlying HBV-related cirrhosis who suffered from acute decompensation. A large Chinese study estimated that the overall ACLF incidence rate over a 10-year period was 2.53 per 100,000 of the general population per year. The short-term mortality of HBV-associated ACLF is high, with 28-day mortality ranging from 40% to 50% depending on the diagnostic criteria as well as class and grade of ACLF. HBV infection was the most common acute insult precipitating HBV-associated ACLF in close to 60% of cases according to published data. The Asia-Pacific Association for Study of Liver ACLF Research Consortium (AARC) reported that acute viral hepatitis A and E contribute to 12.6% of acute insults, whereas a more recent study from the same group in ACLF revealed that complementary and alternative herbal medicines were the commonest cause for drug-induced ACLF [179,180].

In HBV-related ACLF, viral factors were found to have strong association with the development of the catastrophic syndrome. The HBV basal core promoter/precore mutations such as T1753V, A1762T, G1764A, A1846T, G1896A, and G899A correlated with an increased risk of HBV-related ACLF which was supported by the fact that ACLF patients had distinct quasi-species characteristics and higher complexities and diversification within the precore/core gene [181].

A genome-wide association study identified HLA-DR and rs3129859*C allele as the major locus for susceptibility to HBV-related ACLF. This allele was associated with prolonged prothrombin time, faster progression to ascites development and higher 28-day mortality in HBV-ACLF. The authors concluded that the HLA class II restricted CD4+ T-cell pathway on the immunopathogenesis of HBV-related ACLF [182]. Some studies have also shown that HBV genotype B was more susceptible to developing ACLF while this has been refuted in a large metanalysis [183].

Prognosis of HBV-ACLF can be ascertained by a variety of scoring systems which include the standard MELD and MELD-sodium (MELD-Na) scores, EASL chronic liver failure (CLIF)-Consortium-ACLF score (CLIF-C ACLF, better prognostic tool than MELD), integrated MELD, which includes hepatic encephalopathy and age, with an improved sensitivity of approximately 70-80% and the recently proposed AARC score, which integrates bilirubin, creatinine, prothrombin time, lactate, and hepatic encephalopathy which was found to be superior to the MELD in predicting outcomes [177,179,184]. Novel biomarkers such as serum M30 and M65 antigen and Golgi protein 3 - cell death markers were found to predict mortality in patients with HBV-ACLF. However, these have low sensitivity and are not routinely available for use [177,179]. Recently, multiple prediction models were devised by various authors looking at outcomes in patients with HBV-ACLF. One group found that hepatic encephalopathy, neutrophil percentage and platelet levels were independent risk factors for predicting the prognosis of HBV-ACLF. A new prediction model LR(p) was found to have better prediction accuracy than MELD, MELD-Na, and albumin-bilirubin (ALBI) scores [185]. In another study, multivariate analysis indicated that red cell distribution width, neutrophil-to-lymphocyte ratio (NLR), total bilirubin, serum creatinine and international normalized ratio (INR) were identified as risk factors for 90-day mortality in patients with HBV-ACLF. A risk assessment model, called the RNTIC, with cut-off value of 3.08 (sensitivity: 77.89%, specificity: 86.04%) was found to be more predictive of prognosis than MELD, MELD-sodium and Child-Pugh scores [186].

The NLR was also found to be an independent predictor of mortality in patients with HBV-ACLF undergoing treatment with artificial liver support systems (ALSS; combined plasma exchange and bilirubin adsorption performed with continuous renal replacement therapy machine and bilirubin absorbent column) suggesting that liver function in most patients with baseline NLR ≤3 recovered with ALSS treatment, and those with NLRs >6 require emergency liver transplantation [187]. The Chinese Group on the Study of Severe Hepatitis B (COSSH) found that regardless of the presence of cirrhosis, patients with HBV, total bilirubin ≥12 mg/dL and INR ≥1.5 should be diagnosed with ACLF. The COSSH prognostic score (0.741×INR+0.523×HBV-SOFA+0.026×age+0.003×TB) for short-term mortality was superior to five other scores based on both discovery and external validation studies [188]. Additionally, the HINT score, a novel prognostic score based on hepatic encephalopathy, INR, neutrophil count, and thyroid-stimulating hormone (TSH), was simpler and superior to the Child-Pugh, MELD, CLIF-SOFA, and CLIF-C ACLF scores and at least comparable with the COSSH-ACLF score. Sequential measurement of TSH was also helpful in prediction of poor outcomes in HBV-ACLF patients [189].

The age-bilirubin-international normalized ratio-creatinine (ABIC) score >9.44 was superior to the MELD score in predicting short-term survival (one and three month) in HBV-ACLF patients [190]. Another Chinese study found that the plasminogen (significantly lower in HBV-ACLF non-survivors than in survivors) was a good prognostic biomarker and sequential plasminogen measurements help identify clinical course of HBV-ACLF. A new score, known as “the P5”, incorporating plasminogen levels, hepatic encephalopathy occurrence, age, INR and total bilirubin, was significantly superior to the Child-Pugh, MELD and CLIF-C ACLF scores [191]. A study evaluating the ‘regenerating’ ability of the liver showed that overall survival rate within 180 days was 43.48%, and log10-AFP (alfafeto protein) ≥ 2.04 indicated a better prognosis with 76.9% specificity and 62.5% sensitivity for patients with HBV-related ACLF. A new prognostic model called the TACIA score (including total bilirubin, age, creatinine, INR and AFP) was found to predict short-term outcomes in patients with HBV-ACLF in that, patients with lower TACIA scores (<4.34) survived longer [192]. A Chinese group found that low AFP (log value <4.18) was associated with worse prognosis in patients with HBV-ACLF treated with liver support devices and a new model containing AFP, called ALSS‐prognosis model (APM - log value of AFP in microgram/L, INR, bilirubin, age, grade of encephalopathy and serum sodium), which showed potentially better prediction performance than MELD, MELD‐Na, and CLIF‐C ACLF score for short‐term outcomes [193]. A collaborative study on HBV-ACLF utilized the classification and regression tree (CART) analysis to group patients into low and high risk. CART analysis identified three factors prognostic of survival: hepatic encephalopathy, prothrombin time and total bilirubin level; and two distinct risk groups: low risk (28-day mortality, 10.2-39.5%) and high risk (63.8-91.1%). The CART model showed that patients lacking HE and with a prothrombin time ≤ 27.8 s and a bilirubin ≤5 mg/dl experienced less 28-day mortality after ALSS therapy. For HBV-ACLF patients with HE and a PT > 27.8 s, mortality was higher. The authors concluded that, for HBV-ACLF patients at high risk, unnecessary ALSS should be avoided [194]. The World Gastroenterology Organization (WGO) proposed classification according to the underlying liver disease: type A ACLF (patients with underlying non-cirrhotic chronic liver disease), type B ACLF (patients with previous compensated cirrhosis) and type C ACLF (patients with previous decompensated cirrhosis) was utilized to derive a new type-based prognostic model for HBV-related ACLF. Named the “model of ACLF prognosis based on type” or MAPT, the score, developed according to Cox proportional hazards multivariable analysis, included type of ACLF (A, B or C), age, total bilirubin, creatinine, INR and presence or absence of hepatic encephalopathy. The authors found MAPT to be superior to the CLF-C-ACLF, MELD and Child-Turcotte-Pugh scores in predicting 90-day mortality, with an area under the receiver operating characteristic curve of 0.802 with sensitivity of 71.77%, and specificity of 75.82% [195]. Recent high-quality studies have shown that TDF was superior to entecavir in HBV-ACLF (white blood cell count and HBV DNA reduction at two weeks independently predicted mortality at three months); ALSS treatment improved short-term survival and was associated with lower short-term death in patients with HBV-ACLF class 2; corticosteroid treatment did not improve transplant-free survival in patients with HBV-ACLF but, a metanalysis showed that it was effective in reducing jaundice, in-hospital mortality and ascites events; while a prospective multi-center clinical trial showed methylprednisolone therapy (1.5mg/kg/d day 1-3; 1 mg/kg/d day 4-5; and 0.5mg/kg/d day 6-7) increased six-month survival [196-200]. To summarize, specific mutations in HBV predispose to reactivation of the virus leading to ACLF in patients with HBV-related ACLF, which is also governed by HLA susceptibility and virus genotype in certain patient populations. Apart from the classical prognostic scores such as MELD and CLIF scores, newer prognostic tools like AARC, COSSH, ABIC, P5, MAPT and TACIA scores allow the clinician to identify patients who would benefit from early liver transplantation. Furthermore, the ALSS-prognosis model and the CART model help in identifying patients who would fail extracorporeal liver support therapy, in whom early liver transplantation is warranted. Improvement in decisions for clinical management, in the form of prediction and prognostic models and tools for assessing futility and early liver transplantation for HBV-ACLF, have become an important aspect other than the standard antiviral therapy regimen in this difficult to manage group of patients. Further clinically oriented studies and improved understanding of the virus biology and novel modifiable host factors will help the clinician in improving patient care for HBV-ACLF through an algorithmic approach that may become standard of care in the future.

## Conclusions

Our understanding of the structure, biology, viral and immunopathogenesis in chronic HBV-related hepatitis has come a long way. Nonetheless, knowledge gaps still persist that currently limit our therapies toward a complete cure from this globally burdening disease. With the advent of new technologies and better tools such as next-generation sequencing, genome-wide association studies, single-cell RNA sequencing, gene editing and rigorous and well-coordinated collaborative clinical trials, we now understand viral and host-related factors in disease development and progression better than before. Novel modalities of treatments, such as viral RNA interference molecules, capsid assembly blockers, immune checkpoint inhibitors, HBsAg and cccDNA generation blocking molecules and innate immune system modulators, are in the pipeline and will eventually help us improve HBV-related patient outcomes.
